# Inducing metamorphosis in the irukandji jellyfish *Carukia barnesi*

**DOI:** 10.1038/s41598-022-12812-2

**Published:** 2022-05-31

**Authors:** E. O’Hara, J. Seymour

**Affiliations:** Australian Institute for Tropical Health and Medicine, James Cook University, 1/14-88 McGregor Road, Smithfield, QLD 4878 Australia

**Keywords:** Ecophysiology, Biochemistry, Ecology

## Abstract

Here we utilize chemical ecology as a tool to manipulate the biological system of a small, but highly venomous to humans, cubozoan jellyfish, *Carukia barnesi.* We trialled a range of chemical reagents including indole compounds, 9-cis-retinoic acid and lugols solution to induce metamorphosis between the polyp and medusa life stages. An optimum method was determined resulting in a 90% metamorphosis rate to healthy medusa by exposing the polyps to 1 μM of 5-methoxy-2-methylindole for 24 h. Of note is that chemical exposure time significantly impacts health and metamorphosis rates in this species. We also present a theoretical mechanism for the chemical/biological interactions occurring during metamorphosis. This is a significant methodological advancement which now enables rearing of this animal *en mass* in aquaria—a world first for this species—which will subsequently supply and facilitate venom research into this understudied jellyfish.

## Introduction

Irukandji syndrome is the complex and excruciatingly painful condition resulting from a sting from a range of small, almost invisible box jellyfish species. The species most commonly associated with Irukandji syndrome from Northern Australia is *Carukia barnesi*^1,2^, which is known to cause a range of symptoms from extreme muscle pains, nausea and vomiting, to instances which induce intracerebral haemorrhage and death^3,4^. Whilst often revered in the media as one of the most venomous jellyfish in the world^5^, the venom of this animal remains poorly understood, which poses a major detriment to human health and tourism.

The supply of venom may be considered the biggest hindrance to advancing research on this dangerous jellyfish. Current venom collection from this species is reliant on extraction from wild caught adult medusa, collection of which is restricted to what is colloquially termed “stinger season”: the months of approximately November to May when the medusae are present in the wild. *Carukia barnesi* is a logistically difficult species to field sample which further limits the venom supply through large catch variability and no guarantee of sample sizes. Captured individual medusa, even at adult maturity, typical measure less than 12 mm in interpedalial distance, resulting in extremely low venom yield per jellyfish. These factors combined ensure the process of venom acquisition from *C. barnesi* is extremely expensive and logistically difficult. With such a limited supply of venom, research is ultimately finite and very costly, posing a major problem for such a medically important species.

The resolution to this quandary may be to circumvent the use of wild caught medusa entirely and rear these medusa to adulthood *en mass* in aquaria. Mass culture of this venomous jellyfish may be the starting point to facilitating further venom research, an important step towards advancing biomedical, biotechnology and bioprospecting sectors. However, this firstly requires us to step back through the life stages to better grasp how to control them. Three out of the four classes of cnidaria transition from a sessile polyp into a free swimming medusa as part of their life cycle. Each class typically changes in one of three ways as defined by Holstein and Laudet^6^: polyps may undergo strobilation—this occurs in scyphozoans (true jellyfish) in which transverse fissions transform the entire polyp into multiple disc-like young medusa. Polyps may complete metamorphosis, this can occur in cubozoans (box jellyfish) in which the polyps metamorphose into a single medusa, or the polyps may generate medusa though lateral budding^6^ which occurs in hydrozoans. However, some cubozoa have been shown to transition via monodisc strobilation, almost a hybridization of strobilation and metamorphosis in which the polyp is regenerated before a single medusa detaches^7^, which has previously been observed in *C. barnesi*^8^*.* All venom and sting research on *C. barnesi* to date has been derived from the larger adult or sub-adult medusae^9–14^, with no venom analysis conducted on the polyp stages or smaller (< 8 mm^9^) medusas, so it remains a mystery if these very early life stages are as lethal as their adult counterparts. This knowledge gap is predominantly due to difficulties in acquiring these early stages in the wild and extracting sufficient quantities of venom.

Methods to induce metamorphosis/strobilation in jellyfish polyps have been described for multiple species, however the majority of literature is based on scyphozoans, with very little analysis of cubozoans^15,16^. Promising reagents that have successfully been used to induce metamorphosis in other cubozoan species include a number of indole containing compounds^15,16^ which may work as a proxy for a specific peptide sequence upregulated during the metamorphosis process^17^. Lugols solution (aqueous iodine) can successfully induce strobilation in a range of scyphozoan species^18–20^ by influencing an oxidant defense system^20^ and has now been seen to anecdotally produce metamorphosis in cubopolyps (JCU aquarium staff, S. Turner, personal communication, 2020). In addition, retinoids can influence metamorphosis in Hydractinian polyps^21^ which may be applicable to cubopolyps as other cubozoan species have been shown to contain a retinoic receptor^22^.

Populations of *C. barnesi* polyps, created from the in vitro fertilisation of spawning adults, are available in culture^8^. Whilst the natural environmental cue to trigger metamorphosis from polyp to medusa in this species remains a mystery, this current work aimed to artificially chemically induce metamorphosis to create a reliable and increased supply of medusa to facilitate an unrestricted and inexpensive yield of venom for future research.

In this study we explore the following hypothesis: H_0_ Metamorphosis in *Carukia barnesi* polyps is not induced by exposure to chemical compounds, the concentration of said chemicals or the duration of exposure, nor is there a three-way interaction between the three factors.

Available literature documents a number of known metamorphic responses exhibited by this species, consequently allowing us to choose four key stages of metamorphosis to analyse in response to chemical, concentration and time. Tentacle migration is one of the first visually obvious stages of metamorphosis in this species^8^, so was chosen to indicate the initiation of metamorphosis. The polyp producing a detached medusa is quintessentially the very definition of metamorphosis so was chosen as key metric of complete metamorphosis. We also opted to expand on the latter as the literature suggests some chemical compounds can cause deformed unhealthy medusa when inducing metamorphosis^15^ and we needed a realistic metric of healthy detached medusa. Similarly, some chemicals are known to inhibit polyp reformation post metamorphosis^15^, thus, we also chose polyp survival as a metamorphic response to give us an idea of whole colony impact.

## Results

### Primary trials

#### Tentacle migration

**Figure 1:**
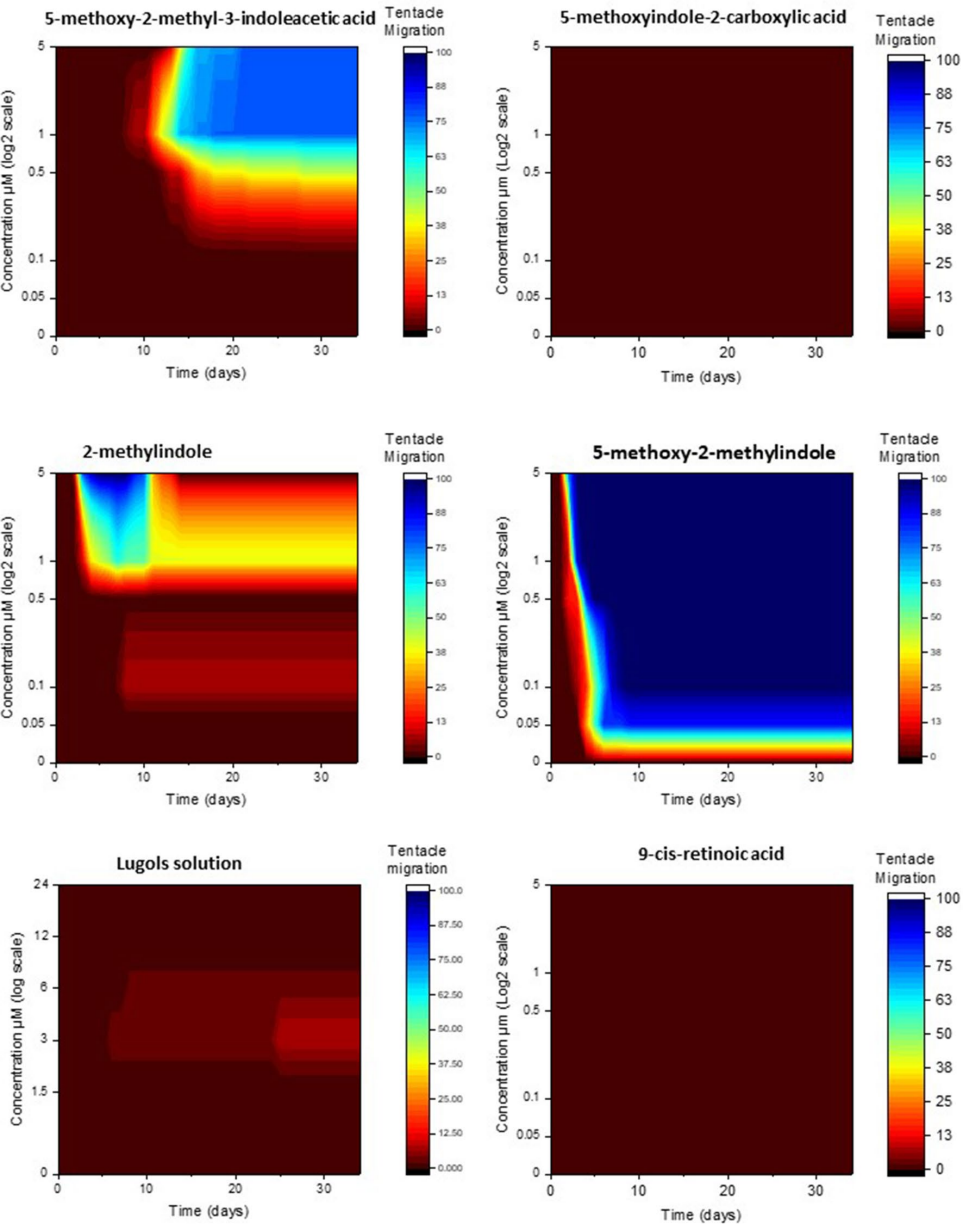
The cumulative percentage of *Carukia barnesi* polyps that displayed tentacle migration after exposure to six chemical treatments, over time in response to chemical concentration.

There was a significant interaction effect of chemical, concentration and time on the mean percentage of polyps displaying tentacle migration (repeated measures three-way ANOVA, *F*(59.922, 345.152) = 9.431, *P* < 0.001). The chemicals 5-methoxyindole-2-carboxylic acid and 9-cis-retinoic acid did not induce any tentacle migration regardless of concentration or time of exposure, whereas 5-methoxy-2-methyl-3-indoleacetic acid, 2-methylindole, 5-methoxy-2-methylindole and lugols solution all induced tentacle migration at varying levels dependant on concentration and time (Fig. 1). The highest concentrations (5 and 1 μM) of 5-methoxy-2-methyl-3-indoleacetic acid induced the highest levels of migration in less time than medium concentrations (0.5 μM) which induced less migration after a longer time, whilst the lowest concentrations (0.5 μM and control) did not induce any migration regardless of time (Fig. 1). The highest concentrations (5 and 1 μM) of 2-methylindole induces nearly 100% tentacle migration between 3 and 5 days, however this migration is reversed completely in the highest concentration 5 μM after approx. 10 days, with the 1 μM concentration reverting to ≈40% migration in a similar time frame. The medium concentration (0.5 μM) begins to induce metamorphosis later at around 9 days, to a much lower percentage, again with the lowest concentrations (0.5 μM and control) never inducing any migration (Fig. 1). 5-methoxy-2-methylindole induced migration at all concentrations, however the higher the quicker migration was induced (Fig. 1). The lugols solution however displays no time/concentration relationship, with low amounts of migration induced only at lower concentrations, induced between 6 and 25 days.

#### Detached medusa

**Figure 2:**
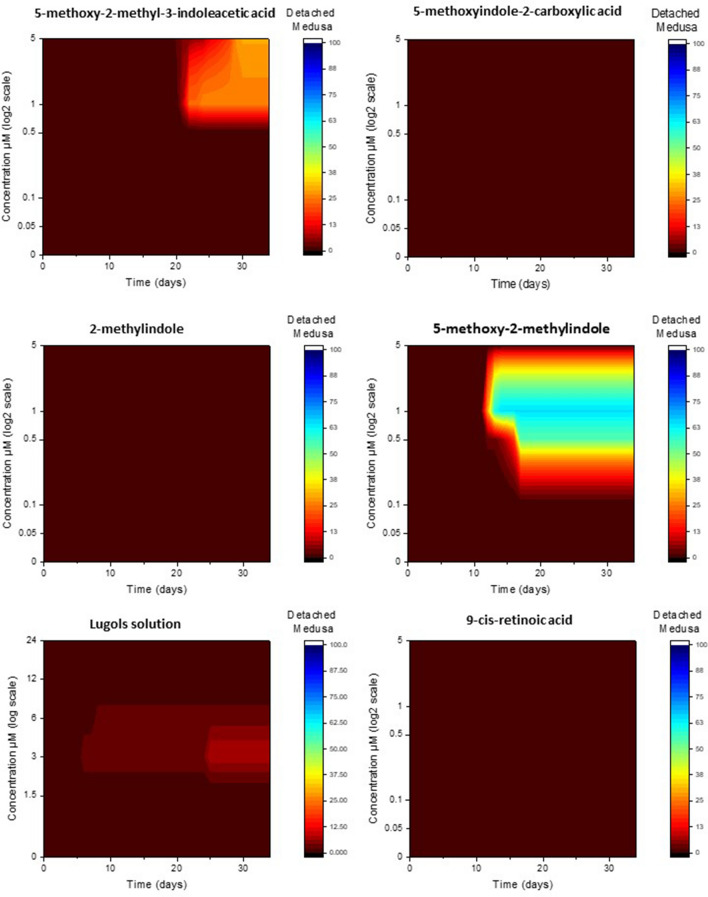
The cumulative percentage of detached medusa from *Carukia barnesi* polyps after exposure to six chemical treatments, over time in response to chemical concentration.

There was a significant interaction effect of chemical, concentration and time on the mean percentage of polyps that produced detached medusa (repeated measures three-way ANOVA, *F*(42.045, 242.177) = 10.854, *P* < 0.001). The chemicals 5-methoxyindole-2-carboxylic acid, 2-methylindole and 9-cis-retinoic acid did not induce any detached medusa regardless of concentration or time of exposure (Fig. 2), despite the fact that 2-methylindole did induce tentacle migration (Fig. 1). Detached medusa were produced in low numbers after exposure to 5-methoxy-2-methyl-3-indoleacetic acid after a long time frame of 20+ days and only at high concentrations, whereas 5-methoxy-2-methylindole produced detached medusas much quicker after only 10+ days with medusa peaking at 1 μM, with fewer to none produced at higher and lower concentrations (Fig. 2). The lugols solution displays no time/concentration relationship, with very low amounts of detached medusa induced only at lower concentrations (Fig. 2).

#### Healthy detached medusa

**Figure 3:**
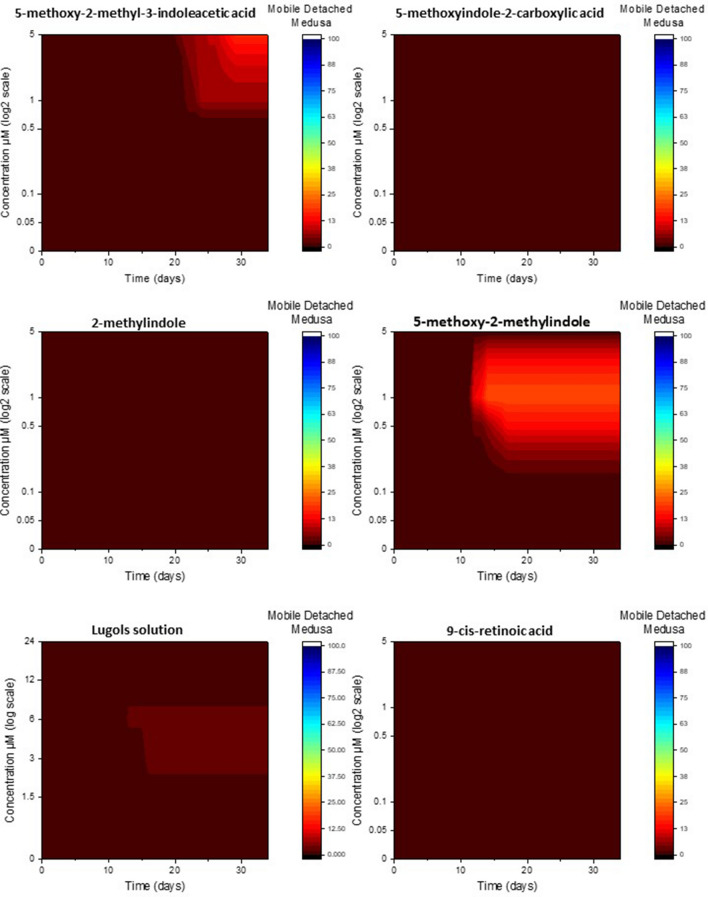
The cumulative percentage of *Carukia barnesi* polyps that detached healthy medusa after exposure to six chemical treatments, over time in response to chemical concentration.

There was a significant interaction effect of chemical, concentration and time on the mean percentage of polyps that produced detached medusa (repeated measures three-way ANOVA, *F*(46.944, 270.396) = 3.486, *P* < 0.001). The chemicals 5-methoxyindole-2-carboxylic acid, 2-methylindole and 9-cis-retinoic acid did not induce any healthy detached medusa regardless of concentration or time of exposure (Fig. 3) which was only to be expected as neither of these produced any detached medusa. Production of healthy detached medusa from 5-methoxy-2-methyl-3-indoleacetic acid was after a long time frame of 20+ days and only at high concentrations (Fig. 3), the same pattern as the production of detached medusa (Fig. 2) except the healthy medusa were at lower quantities (Fig. 3). Again similar to the production of detached medusa 5-methoxy-2-methylindole produced healthy detached medusas much quicker after only 10+ days peaking at 1 μM but percentages of healthy medusa were lower than those of just detached medusa (Figs. 2, 3). Lugols solution produced very low amounts of healthy detached medusa which were induced only at lower concentrations, however unlike all other chemicals, 100% of the detached medusa were also healthy.

#### Survival of polyps that did not morph

**Figure 4:**
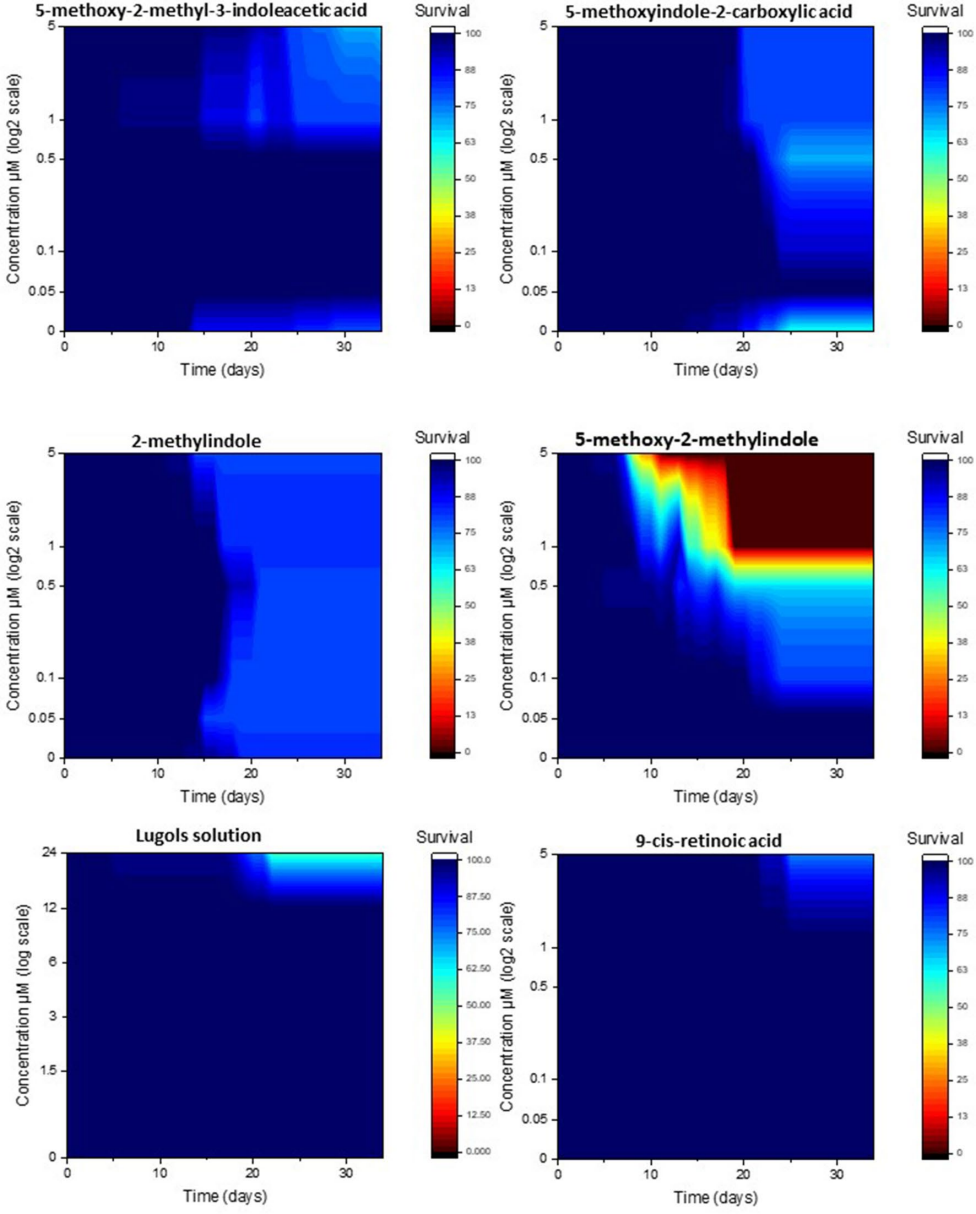
The cumulative percentage of *Carukia barnesi* polyps that survived (that did not morph) after exposure to six chemical treatments, over time in response to chemical concentration.

There was a significant interaction effect of chemical, concentration and time on the mean percentage of polyps that produced detached medusa (repeated measures three-way ANOVA, *F*(38.857, 223.814) = 2.062, *P* < 0.001). All chemicals caused some polyp mortality, which with the exclusion of 2-methylindole, was concentration dependent. 5-methoxy-2-methyl-indoleacetic acid, lugols solution and 9-cis-retinoic acid all had increased polyp mortality at high concentrations typically only towards the end of the 34 day experiment. 5-methoxyindole-2-carboxylic acid also induced mortality towards the end of the experiment time, but had the highest mortality rates at the medium concentration of 0.5 μM (Fig. 4). 2-methyindole was the only chemical to cause a consistent rate polyp mortality (approx. 80% survival) at all concentrations. 5-methoxy-2-methylindole was the only chemical to cause 100% of the polyps that had not morphed to die, a clear concentration/time relationship is evident. The higher the concentration the quicker it causes polyps to die (Fig. 4). Note, low levels of mortality are evident in some of the controls (concentration 0), suggesting there is some low level natural mortality in these experimental conditions.

### Optimisation trial

**Figure 5:**
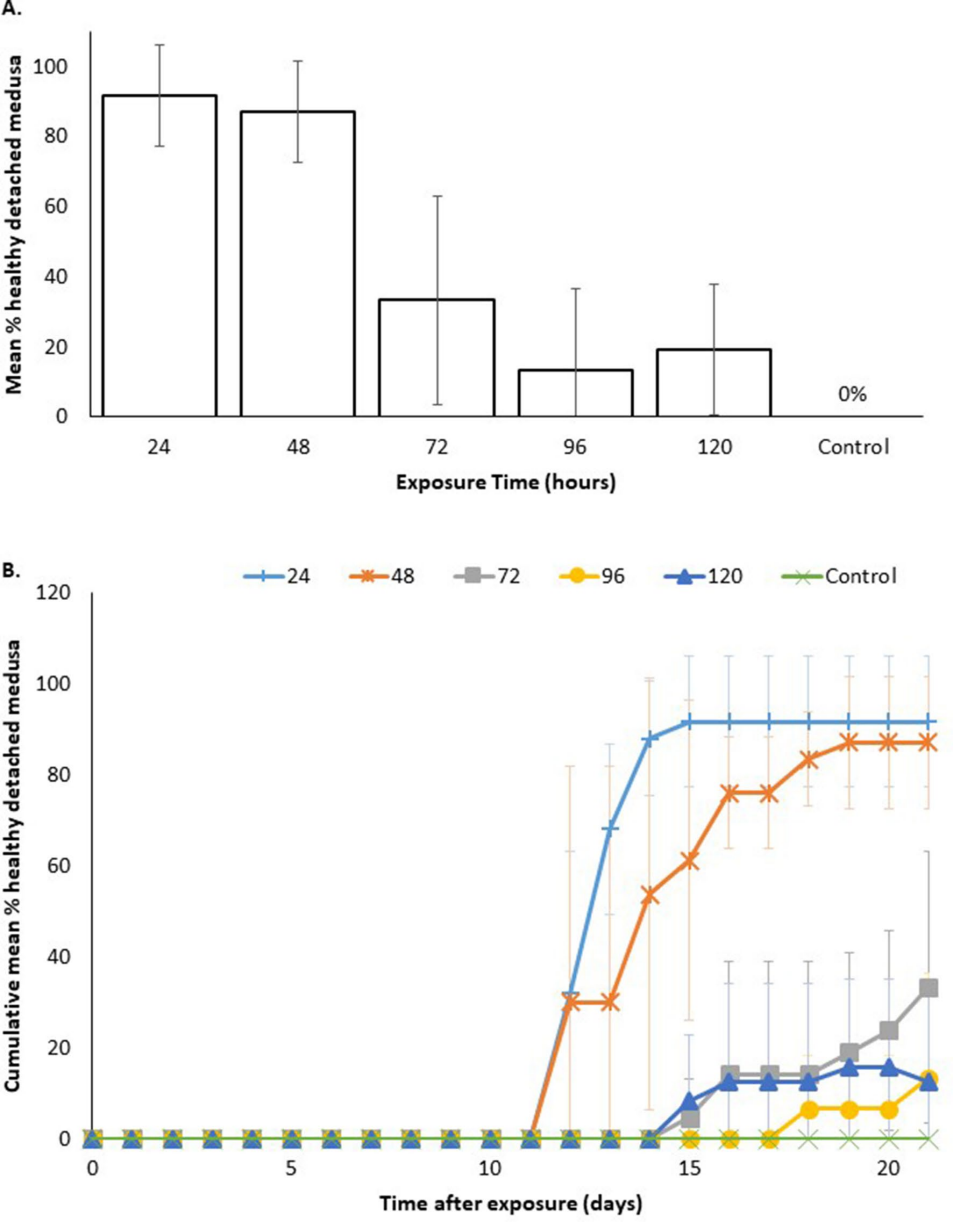
(**A**) Total Mean percentage (± SD error bars) after 21 days of healthy medusas produced by *Carukia barnesi* polyps exposed to 5-methoxy-2-methylindole at 1 μM for different time periods. Exposure time (hours) before solution was changed out to seawater only. Control is seawater only. (**B**) Cumulative mean percentage (± SD error bars) of detached healthy medusas over a 21 day period following exposure to 5-methoxy-2-methylindole at 1 μM.

5-methoxy-2-methylindole at 1 μM was the peak chemical and concentration deduced from primary trials, resulting in around 20% healthy detached medusa and thus was chosen as the sole chemical for use in the optimisation trial. There was a statistically significant difference in the mean percentage of healthy medusas produced by polyps at different exposure times to 5-methoxy-2-methylindole at 1 μM (ANOVA, F = 12.631, df = 5, 12, *P* < 0.05) (Fig. 5A). Post hoc Tukey pairwise comparison (*P* = 0.05) showed that there was no significant difference between 24 h (mean = 91.7 ± 14.4%) and 48 h treatments (mean = 87.1 ± 14.5% SD). There was no significance difference between 72 h (mean = 33.3 ± 29.7% SD), 96 h (mean = 13.3 ± 23% SD), 120 h (mean = 19.2 ± 18.8% SD) and control (mean = 91.7 ± 14.4%) treatments.

There was however overall a significant negative effect of exposure time and the percentage of healthy medusa produced. Percentage of healthy medusa detached was highest for 24 and 48 h, which were both significantly higher than the 72, 96, 120 h treatments and the control.

The optimisation trial resulted in a peak of 90% healthy detached medusa, when the polyps are exposed to 5-methoxy-2-methylindole at 1 μM for 24 h, then changed to fresh seawater (Fig. 5A). Over 21 days of observations post exposure, the 24 and 48 h treatments produced the largest percentages of healthy detached medusa in the quickest time frames, with the longer the exposure times taking longer to produce less medusa (Fig. 5B).

## Discussion

This study aimed to ascertain an efficient method to artificially induce metamorphosis from polyp to medusa in the cubozoan *Carukia barnesi*. The intention was to verify a chemical and concentration combination that had the capacity to produce the highest quantities of healthy medusa, to facilitate a plentiful supply of medusa for venom collection and further research. Indoles have been successfully used to induce metamorphosis in a small number of cubozoan species^15,16^, however high concentrations have been reported to cause severe deformities and inconsistent metamorphosis^15^. As such we placed an emphasis on quantifying both the total of detached medusas and the further subset of those that were healthy, fully formed and actively mobile, as that was the most useful metric to enable subsequent rearing of the medusae to adults. Both 5-methoxy-2-methyl-3-indoleacetic acid and 5-methoxy-2-methylindole induced medusa formation and detachment, with the amount of healthy medusa produced much lower than the total number detached. Those that were not healthy presented deformities previously noted in the literature^15^ including shriveled bells and the inability to swim, rendering them unsuitable for further rearing. Lugols solution was the only chemical in which all medusa that formed and detached were healthy, though induction of metamorphosis was much more inconsistent and at lower quantities than the two successful indoles.

Previous assays of indoles on the cubozoan *Carybdea* sp. documented severe medusa and phenotypic deformities theorised to be toxicity associated when dosed at high concentrations (50 and 20 μM)^15^. From preliminary trials we deduced that the indole concentrations employed in the literature^15,16^ that induce metamorphosis in other cubozoan species were completely unsuitable for our species *Carukia barnesi.* 5-methoxy-2-methylindole caused total mortality of *C. barnesi* polyps within 2–3 days, when added at 50 μM^15,16^ and 20 μM^15^, thus, our experimental range was reduced to 0.05–5 μM to negate this toxicity effect. However, we did still note higher mortality rates in higher concentrations. 100% ethanol was used to dissolve the indoles into solution, before being diluted to experimental concentrations with sea water as per the literature; where this seems suitable for other cubozoan species^15,16^, our carrier solution (100% ethanol) controls also caused mortality at higher concentrations in preliminary tests—suggesting *C. barnesi* species has high chemical sensitivity in comparison to other species. As such, we reduced the concentration of ethanol to 50% to reduce toxicity of the carrier solution whilst still maintaining its efficiency as a solvent. Some natural variability in survival was evident, as slight mortality was observed in some controls. This could be natural, or could be a result of experimental caveats such as small water volumes.

Of the six chemicals tested three successfully induced metamorphosis in *C. barnesi* with varying efficacy: 5-methoxy-2-methylindole, 5-methoxy-2-methyl-3-indoleacetic acid and lugols solution (from highest to lowest percentage of metamorphosis). With the two indoles, a correlation is evident in that the most successful chemical/concentration combination for metamorphosis also presents the highest levels of polyp mortality (Figs. 2, 5), suggesting that they may act to induce metamorphosis by stressing polyps and simultaneously are toxic to the polyps. Potentially an extension of this toxicity, it has previously been noted that indoles can inhibit polyp reformation after medusa production^15^, thus we elected to measure survival of polyps that did not metamorphose. *Carukia barnesi* does usually leave a polyp remaining after producing a single medusa^8^, arguably more similar to monodisc strobilation observed in scyphozoans than the complete metamorphosis of one whole polyp into one single medusa which is described in cubozoans. In a most unusual occurrence, 5-methoxy-2-methylindole not only induced metamorphosis in the adult polyp, but also in any lateral buds growing from the adult polyp. This documents, to our knowledge, the first incidence of a single cubozoan polyp undergoing multiple metamorphoses. This is of course assumed to be a forced measure from artificial stimulation and not propounded to be an in situ characteristic of this species.

**Figure 6:**
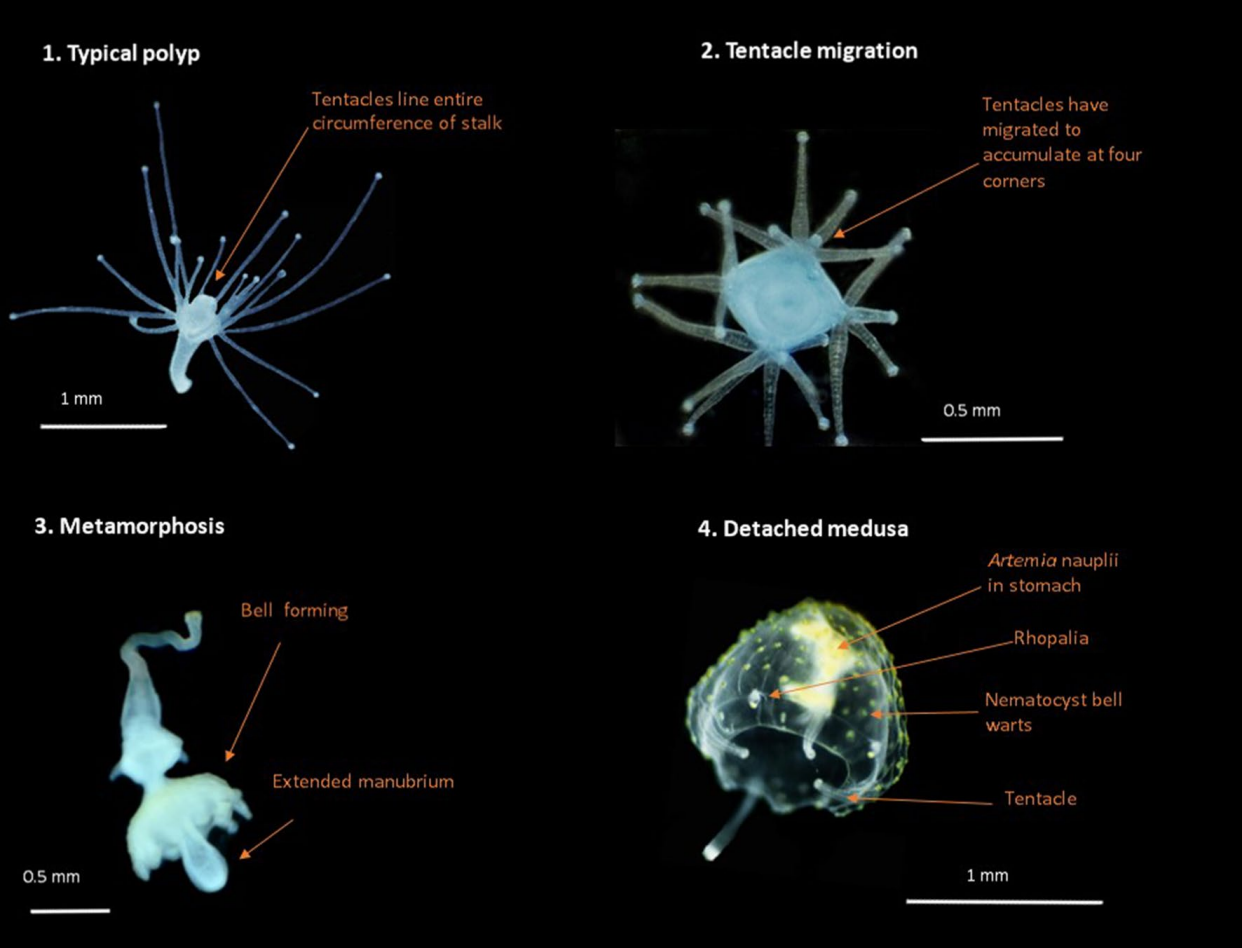
Representative stages observed in metamorphosis of *Carukia barnesi* polyps. (1) Typical healthy polyp (side view). (2) Polyp exhibiting the first stage of metamorphosis, tentacle migration (dorsal view). (3) Medusa forming on the end of a polyp during metamorphosis. (4) A medusa detached from polyp following metamorphosis. Note tentacles are not fully formed at this immature stage.

The chemicals explored in this work were intended to be used as a tool to induce metamorphosis and do not illustrate natural triggers. Some *Aurelia* jellyfish upregulate a specific peptide during strobilation, which contains indole rings of two tryptophans^17^, thus indoles are theorised to essentially shortcut the natural environmental trigger and act internally by replicating a key part of this peptide to artificially induce metamorphosis in other species. Indoleamines have also been linked to endocrine-like signalling and metamorphosis in Cnidarians^23^. Of specific interest, when added exogenously the indoleamine melatonin (a derivative of tryptophan) can initiate expansion of the oral disk and protrusion of the actinopharynx in sea anemones^23,24^, similar to our observation with *C. barnesi* medusa production, in which one of the first changes (subsequent to tentacle migration) was protrusion of the mouth (Fig. 6 (3)). Iodine has long been known to induce strobilation in *Aurelia* jellyfish^18^, with Berking et al.^20^ succinctly linking previous works by Paspalev^25^ and Riley and Chester^26^ to highlight that in sea water containing below average trace amounts of iodine *Aurelia* strobilation is inhibited, suggesting it plays a key role in strobilation. Whilst the iodine and indoles may initially appear to act on polyps in very different ways, there is some crossover in their activity which may speculatively rationalize the capability of two very different chemicals to induce metamorphosis. Iodine induces metamorphosis in *Aurelia aurita* (scyphozoa, Cnidaria) by functioning as an oxidative defence system, in which iodine initially reacts with tyrosine, which subsequently reduces tyrosine levels^20^. Indoleamines such as L-Tryptophan, L-5-hydroxytryptophan and serotonin have been shown to increase tyrosine aminotransferase activity in mice^27^, and whilst tyrosine aminotransferase is usually found in the liver, genes encoding for this have been found in *Aiptasia* (Anthozoa, Cnidaria)^28^. Tyrosine aminotransferase is an enzyme which catalyses tyrosine to 4-hydroxyphenylpyruvate, thus tyrosine levels would drop as the indoleamines increase enzyme activity. Serotonin (an indoleamine) has further been demonstrated to influence metamorphosis in other cnidarian species^29^. Thus, we suggest that there is a theoretical mechanism of action in which both indoles and iodine induce metamorphosis in cnidarian polyps by altering levels of tyrosine.

Using the peak chemical and concentration from the primary (trials 5-methoxy-2-methylindole at 1 μM), we ran a final optimisation trial to deduce if we could reduce the toxicity effect by decreasing the length of exposure time and potentially increase healthy medusa yield. Length of exposure had a significant effect on the quantity of healthy medusa produced by *C. barnesi* polyps, with results peaking at a mean of 90% healthy medusa production when the indole solution was exchanged for seawater after 24 h. Healthy medusa production decreased the longer the polyps were exposed to the solution, with longest exposure levels (96 and 120 h) producing quantities of medusa in line with the quantities seen in the preliminary trials for 5-methoxy-2-methylindole at 1 μM, in which the polyps were exposed to the indole solution for the entire experimental period. For the optimization trials, healthy detached medusa was the only metric collected and analysed, as it is the most valuable measure for the experimental aim. Polyp survival was not measured because as previously noted, we observed the indoles inhibited polyp reformation in line with the observations of Helm and Dunn^15^.

The significant increase in healthy medusa production at 24 and 48 h of exposure alludes to the idea that too much chemical is overwhelming the polyp. In line with the toxicity noted with increased concentration in the preliminary trial, the longer exposure times in the optimization trials saw the polyps take longer to produce less medusa (Fig. 5B). In this artificial set up, the levels of indole in the polyp are being increased exogenously, whereas naturally it is an internally regulated event. From our results, the prime exposure time is 48 h or less, suggesting the internal mechanism that initiates metamorphosis is naturally only switched on for a very short amount of time, as we see longer exposure times or greater concentration is detrimental to the medusae wellbeing. In the wider search for the natural environmental cue, it would then seem prudent to investigate short term events that occur during the stinger season such as marine spawning events, photoperiod and/or wet season pulses such as monsoonal downpours, all of which could physically influence the chemical biology of these polyps.

The implications of this work will enable mass production of *C. barnesi* medusa, which will facilitate a supply of medusae and therefore venom, as and when necessary. For the first time, venom analysis of this newly detached medusa (< 1 mm) may be conducted to elucidate ontogenetic differences, as presently all venom research in this species has been conducted on the larger adult or sub-adult medusas (> 8 mm). Up until now, medusa collection was restricted to labour intensive and expensive field sampling, confined to the stinger season (November–May) which is also accompanied by the bad weather and poor field conditions of the monsoon season. The loss of polyps due to the indoles propensity to inhibit polyp reformation is negligible, as polyp cultures exponentially asexually reproduce, populations should be easily replaced. We have determined an optimum method for inducing metamorphosis in the Irukandji jellyfish *Carukia barnesi,* resulting in a 90% metamorphosis rate to healthy medusa by exposing the polyps to 1 μM of 5-methoxy-2-methylindole for 24 h. These medusa are fit enough to be further reared in aquaria and we hope this increased supply can further research into the venomous nature of this animal.

## Method

### Animal husbandry

*Carukia barnesi* polyps were available in culture from the James Cook University Aquarium, spawned from medusa originally collected near Double Island, North Queensland, Australia (16° 43.5′ S, 145° 41.0′ E) in 2014 and 2015^8^. Populations exponentially increase through asexual reproduction^8^. Detached buds and swimming polyps were collected from the main culture, and transferred into 6-well tissue culture plates in natural filtered seawater. Plates were maintained in darkness to inhibit algae growth at 27 °C in a constant temperature cabinet. Buds and swimming polyps were left to develop and attach to well bottoms, at which point they were then fed freshly hatched *Artemia* nauplii and water changed 2–3 times per week. Lids remained attached to tissue culture plates to negate water evaporation and maintain a stable salinity. Polyps were maintained in this way for a minimum of 4 months before experiments began, with all individuals matured with the ability to asexually reproduce further buds. To preserve water quality^15^ polyps were starved for two days prior to experiment start and were not fed for the duration of the trials. One day prior to the experiment start, all immature buds and polyps were removed from wells, leaving approximately 5–10 mature polyps attached to the substrate for analysis.

### Preparation of reagents

#### Reagents

Six chemicals were trialed in the current study to induce metamorphosis in *C. barnesi* polyps. Four indole containing compounds were chosen that have previously been trialed with other cubozoan species: 5-methoxy-2-methyl-3-indoleacetic acid, 5-methoxyindole-2-carboxylic acid, 2-methylindole^16^ and 5-Methoxy-2-methylindole^15,16^. Along with the retinoic X receptor 9-cis-retinoic acid and lugols solution.

#### Indole compound treatments

Chemical concentrations of indoles documented in the literature were used to conduct preliminary concentration tests. Fifty mM stock solutions were prepared with 100% ethanol, which was diluted with filtered seawater to the desired experimental concentrations: 50 μM^16^, 20 μM and 5 μM^15^. Due to high fatality rates at all of these concentrations when used in this study on *C. barnesi*, all concentrations were diluted. Fifty mM stock solutions of 5-methoxy-2-methyl-3-indoleacetic acid, 5-methoxyindole-2-carboxylic acid, 2-methylindole and 5-Methoxy-2-methylindole were prepared with 50% ethanol (50% Milli-Q® water) and stored at − 20 °C. Fifty mM stock solutions were diluted with filtered seawater to the experimental concentrations of 5 μM, 1 μM, 0.5 μM, 0.1 μM and 0.05 μM. The carrier solution of 50% ethanol (50% Milli-Q® water) was diluted to the equivalent of the experimental concentrations listed above for use as a control, and incorporated into data as concentration 0. Seventeen ml of solution was added to polyps to fill each well of a 6-well plate.

#### Iodine treatment (lugols solution)

Aqueous iodine in the form of Lugols solution (0.37% iodine and 0.74% potassium iodide (sigma product information)) was prepared with equivalent concentrations of moles iodine/iodide: 1.5 μM, 3 μM, 6 μM, 12 μM and 24 μM. Filtered seawater only was used a control for this treatment and incorporated into data as concentration 0. 17 ml of solution was added to polyps to fill each well of a 6-well plate.

#### Retinoid treatment

To reduce ethanol associated fatality of polyps 0.015% ethanol in Milli-Q® water was used to prepare a 1 mM stock solution of 9-*cis*-Retinoic acid. The 1 mM stock solution was diluted with filtered seawater to the experimental concentrations of 5 μM, 1 μM, 0.5 μM, 0.1 μM and 0.05 μM. The carrier solution of 0.015% ethanol (Milli-Q® water) was diluted to the equivalent of the experimental concentrations listed above for use as a control, and incorporated into data as concentration 0. 17 ml of solution was added to polyps to fill each well of a 6-well plate.

### Metamorphosis trials

#### Primary trials

Experimental concentrations of reagents were added to *C. barnesi* polyps growing in the wells of sterile 6-well tissue culture plates. One plate was used per chemical, per concentration, in which five wells functioned as replicates containing the chemical being trialed, whilst the sixth well contained only the control medium. Five concentrations were run for each of six chemicals; 30 plates in total.

The filtered seawater the polyps were growing in was exchanged for the experimental chemical on day 0, and was not changed for the duration of the trial. Lids remained attached to tissue culture plates to negate water evaporation and hence salinity changes.

Polyps in each well were photographed each day through a dissection microscope over a period of 34 days. Results were then recorded from the photographs, categorised (Fig. 6) as the number of polyps which displayed:

*Tentacle migration*: one of the key signs of metamorphosis in this species, polyp tentacles merge, migrating to form four distinct corners in a square shape^8^.

*Detached medusa*: a medusa formed and detached from the polyp, recorded regardless of health.

*Mobile detached medusa*: a healthy medusa formed and detached from the polyp, with the ability to swim.

*Polyp survival*: this was then used to calculate the number of polyps which survived the treatment which did not metamorphose.

#### Optimisation trial

The optimal chemical and concentration was then deduced by choosing the combination that produced the largest percentage of healthy detached medusa, in this case 5-methoxy-2-methylindole at 1 μM. A final trial was then run with this to determine if length of chemical exposure could optimize healthy medusa yield. Three replicates of a minimum of five polyps were used per treatment, in which in 1 μM of 5-methoxy-2-methylindole (in seawater) was added to polyps for 24, 48, 72, 96 and 120 h, before the solution was changed to fresh seawater. A sea water only control was also run. The total number of healthy detached medusa were recorded each day.

### Data analysis

All statistical analysis was conducted in IBM SPSS Statistics Ver28. Graphs were produced in Microsoft Excel 2016 and OriginPro Graphing and Analysis 2021.

#### Primary trials

The effect of chemical, concentration and time was analysed using a repeated measures three-way ANOVA for four sets of data gathered during the metamorphosis process: percentage of polyps to display tentacle migration, percentage of polyps to have medusa detach, percentage of polyps to have healthy swimming medusa detach, percentage survival of polyps that did not metamorphose. Percentage data was arcsine square root transformed prior to analysis. Mauchly's Test of Sphericity indicated that the assumption of sphericity had been violated on all four sets of data and therefore, a Greenhouse–Geisser correction was used.

#### Optimisation trial

Differences in the mean percentage of healthy medusa produced at different exposure times was analysed using ANOVA. Differences between means were elucidated using a Post hoc Tukey pairwise comparison test (Tukey HSD alpha 0.05).

## Supplementary Information

Supplementary Information 1.

Supplementary Information 2.

## Data Availability

The datasets used and/or analysed during the current study are available as supplementary files to this manuscript.
